# Understanding User Needs for an Adaptive System to Support Personal Activities and Reinforce Cognition (SPARC)

**DOI:** 10.1093/geroni/igaf122.1637

**Published:** 2025-12-31

**Authors:** Wendy Rogers, Raksha Mudar, Tracy Mitzner, Sara Czaja, Walter Boot, Neil Charness

**Affiliations:** University of Illinois Urbana-Champaign, Champaign, Illinois, United States; University of Illinois Urbana-Champaign, University of Illinois Urbana-Champaign, Illinois, United States; Person in Design, Atlanta, Georgia, United States; Weill Cornell Medicine, New York, New York, United States; Weill Cornell Medicine, New York, New York, United States; Weill Cornell Medicine, New York, New York, United States

## Abstract

The likelihood of developing a cognitive impairment (CI) increases with age. Older adults with CI experience difficulties performing a range of everyday activities and are at risk for social isolation. Technology-based interventions have the potential to improve everyday functioning. Artificial intelligence capabilities can be harnessed to tailor solutions to the unique and changing needs of older adults with CI. Most prior efforts focused on cognitive training or rehabilitation but have not included other aspects of functioning such as everyday activities or social engagement. Our goal is to develop and evaluate an innovative intelligent adaptive software system to support personal activities and reinforce cognition (SPARC). The system will be designed to adapt to the needs and abilities of the user with CI, following the CREATE model of user-centered design. We started with interviews with subject matter experts to assess older adults’ needs for activity support in their homes and identify potential technology solutions. We interviewed both clinicians and technical experts to obtain guidance for the initial system design. In this presentation, we will present results of focus group interviews with older adults who have CI wherein we present prototype images of SPARC and explore their perceptions of usefulness, ease of use, preferences for instructional support, and ideas for specific content that would be of interest to them. This multi-faceted needs assessment approach will guide design of SPARC to support everyday activities, primarily in the home environment. Our findings inform design of other technologies and interventions to support older adults with CI.

